# Hydrological Impacts of Land Use Change and Climate Variability in the Headwater Region of the Heihe River Basin, Northwest China

**DOI:** 10.1371/journal.pone.0158394

**Published:** 2016-06-27

**Authors:** Ling Zhang, Zhuotong Nan, Yi Xu, Shuo Li

**Affiliations:** 1 Cold and Arid Regions Environmental and Engineering Research Institute, Chinese Academy of Sciences, Lanzhou, 730000, China; 2 University of Chinese Academy of Sciences, Beijing, 100049, China; 3 Key Laboratory of Virtual Geographic Environment, Ministry of Education, Nanjing Normal University, Nanjing, 210023, China; 4 Jiangsu Center for Collaborative Innovation in Geographical Information Resource Development and Application, Nanjing, 210023, China; 5 State Key Laboratory Cultivation Base of Geographical Environment Evolution, Jiangsu Province, Nanjing, 210023, China; University of Vigo, SPAIN

## Abstract

Land use change and climate variability are two key factors impacting watershed hydrology, which is strongly related to the availability of water resources and the sustainability of local ecosystems. This study assessed separate and combined hydrological impacts of land use change and climate variability in the headwater region of a typical arid inland river basin, known as the Heihe River Basin, northwest China, in the recent past (1995–2014) and near future (2015–2024), by combining two land use models (i.e., Markov chain model and Dyna-CLUE) with a hydrological model (i.e., SWAT). The potential impacts in the near future were explored using projected land use patterns and hypothetical climate scenarios established on the basis of analyzing long-term climatic observations. Land use changes in the recent past are dominated by the expansion of grassland and a decrease in farmland; meanwhile the climate develops with a wetting and warming trend. Land use changes in this period induce slight reductions in surface runoff, groundwater discharge and streamflow whereas climate changes produce pronounced increases in them. The joint hydrological impacts are similar to those solely induced by climate changes. Spatially, both the effects of land use change and climate variability vary with the sub-basin. The influences of land use changes are more identifiable in some sub-basins, compared with the basin-wide impacts. In the near future, climate changes tend to affect the hydrological regimes much more prominently than land use changes, leading to significant increases in all hydrological components. Nevertheless, the role of land use change should not be overlooked, especially if the climate becomes drier in the future, as in this case it may magnify the hydrological responses.

## Introduction

It is widely agreed that land use change and climate variability are two active environmental factors profoundly affecting watershed hydrology [1–3]. Land use changes, which are mostly induced by human activities, affect hydrological processes such as evapotranspiration (ET), interception and infiltration, resulting in alterations of surface and subsurface flows [3, 4]. Climate variability also brings distinct changes to hydrological regimes and influences spatial and temporal patterns of water resources in a region [5]. It was reported in the Fifth Assessment Report of the Intergovernmental Panel on Climate Change [6] that a warming climate in the past half century has occurred in almost every region across the world, especially in the mid-latitudes of the northern hemisphere [7]. In the context of global climate change, most of China has also experienced a significant warming trend over the past decades and the trend is expected to continue in the future [8, 9]. Nevertheless, there is no a clear trend in precipitation since 1960 and future projections are subject to high uncertainty [8]. The changes in both temperature and precipitation exhibit remarkable regional variability; the northwest China is projected to experience a warmer and wetter climate in the future [10]. The rising temperature accompanied with altered precipitation patterns and intensity may have substantial hydrological consequences such as accelerating the hydrological cycle and increasing the frequency of occurrence of hydrological extremes, particularly in the arid regions of northwest China where the scarcity of water resources heavily constrains the local agricultural and economic developments [11–13].

In recent years, many studies have been conducted to examine hydrological impacts of land use change and/or climate variability, in which their roles have been identified [3, 12–20]. The findings of these studies are helpful for understanding the causes of hydrological variations as well as developing adaptation measurements. However, the combined effects of land use change and climate variability and their individual contributions are still not fully understood and it provides motivation for further study [3, 21]. Moreover, the effects may vary from place to place due to their geographical varieties, necessitating further investigation at regional scales [17, 18].

As the second largest inland river basin in the arid region of northwest China, the Heihe River Basin (HRB) is of strategic importance because the Hexi Corridor, which is located across the middle HRB, connects the inland Xinjiang Province with the rest of northern China [22, 23]. It is a typical inland river basin attracting wide attentions of scholars and governmental authorities, owing to its distinct landscape patterns as well as typical and outstanding water problems. The basin is composed of three sections from south to north: upstream, midstream and downstream. The upper HRB, characterized by mountainous terrains, is the water source area and the headwater region of the Heihe River. The middle HRB, characterized by alluvial plain oasis, is the primary water consumption area where the irrigated agriculture consumes more than 90% of the total water supply [24]. The lower HRB, which is covered by a vast Gobi desert, is the place where the runoff disappears through seepage and ET [25]. Owing to the increasing population, economic growth and expansion of farmland, water consumption in the middle HRB dramatically increased over the period from the 1960s to the 1990s, resulting in an abrupt reduction in water supply to the lower HRB [26]. As a result, the terminal lakes were completely dried up; the loss of biodiversity was accelerated; and the desertification was intensified [25]. In order to mitigate these issues, the China government carried out the Ecological Water Diversion Project (EWDP) in 2000 to ensure a minimum amount of water for ecological use in the lower HRB, by which the eco-environmental conditions have been substantially improved [27]. However, it also leads to more intense competition in water use between regions and sectors as the total available water resource in the middle HRB is decreased. To sustain water resources in the HRB, it is necessary to improve the understanding of hydrological variations induced by land use and climate changes, especially in the headwater region where runoff is generated and maintains the water supply to almost the entire basin [19].

A number of studies have been carried out to explore impacts of land use change and climate variability on hydrological regimes in the HRB and they mainly focus on three issues: (i) past land use changes and their impacts; (ii) past climate changes and their impacts; and (iii) potential hydrological impacts of land use change and climate variability in the future. Some closely related studies are summarized in Table 1. There are obviously some deficiencies: (i) most studies only considered one type of change (either climate or land use), with few on the combined effects; (ii) the investigations were usually conducted through statistical time series analysis rather than hydrological modeling approach, the former of which is not physically explicit and is difficult to quantify the extent to which these changes are due to changes in land use, climate variability, or both; and (iii) the climate or/and land use data have not be updated to the latest. Although some of the existing studies have already considered the combined hydrological impacts, they established climate change scenarios on the basis of general circulation model (GCM) projections [24, 28–30]. For example, based on climatic projections of Hadley Centre Couple Model version 3 (HadCM3), Zhang et al (2015) [29] recently explored the joint impacts of land use and climate changes on streamflow and hydrological extremes in the upper and middle HRB in the future under consistent emission scenarios (A1B and B1). However, few of them have adopted the hypothetical climate scenarios that were established upon the analysis of long-term climate observations, which may seem more reliable than GCM climate scenarios for a relatively small region with highly spatially heterogeneous and complex terrain, such as the upper HRB, owing to the inherent coarse resolution of GCMs and their discrepant projections [28, 31]. Moreover, the impacts of land use and climate changes on the hydrological regimes in the recent past were rarely quantitatively explored using a modelling approach.

**Table 1:** Summary of relevant studies in the HRB (the current paper is added for completeness). In the ‘key results’ column the abovementioned three components are identified by the code: (1) assess past land use changes and their hydrological impacts; (2) assess past climate changes and their impacts; and (3) assess potential hydrological impacts of land use change and climate variability in the future. Not all papers assess the three components and a N/A representing ‘not applicable’ directly follows the code in such cases.

Study	Study area	Observed data / Method	Key results
1. Cai et al. (2014) [32]	Upper HRB	Climate: 1990–2010 / statistical analysis	(1) N/A. (2) Increasing temperature led to rising river flow. (3) N/A.
2. Zhang et al. (2003) [33]	HRB	Climate: 1950–2000 / statistical analysis	(1) N/A. (2) Climate changes caused decreasing runoff in most branches. (3) N/A.
3. Sang et al. (2014) [18]	Upper and middle HRB	Climate: 1960–2000 / wavelet analysis	(1) N/A. (2) Increasing precipitation generated more runoff in both the upper and middle reaches but the effects of temperature varied with region. (3) N/A
4. Wu et al.(2014) [30]	Upper and middle HRB	Climate: 1981–2005, land use: 2010 / hydrological modeling	(1) N/A. (2) N/A. (3) Projected land use changes and climate changes under the RCP 4.5 scenario will change the water yield by −1.8% and +9.8% separately and by +8.5% jointly.
5. Zang et al. (2014) [23]	HRB	Climate: 1958–2010 / hydrological modeling and trend analysis	(1) N/A. (2) Increased blue water and significantly decreased green water were induced by climate change. (3) N/A.
6. Zhang et al. (2015) [25]	HRB	Climate: 1960–2012 / statistical analysis	(1) N/A. (2) Rising temperature and precipitation led to an increase in streamflow in upper HRB. (3) N/A.
7. Hu et al. (2015) [26]	Middle HRB	Land use: 2000, 2007 and 2011 / statistical analysis	(1) Increasing farmland resulted in groundwater overdraft. (2) N/A. (3) N/A.
8. Nian et al. (2014) [34]	Middle HRB	Land use: 1965, 1986 and 2007 / statistical analysis	(1) Increasing farmland induced overuse of surface water and overexploitation of groundwater. (2) N/A. (3) N/A.
9. This study	Upper HRB	Climate data 1960–2014, land use in 1986, 2000 and 2011 / hydrological modeling and scenario analysis	(1) Land use changes induced increase in ET but decline in surface runoff and streamflow. (2) Climate presented a wetting and warming trend and led to rising streamflow and ET. (3) Future land use change and climate variability jointly induce continuous increases in ET and streamflow; and climate change is primarily responsible for hydrological variations.

The objective of this work is therefore to quantify the separate and joint hydrological impacts of land use change and climate variability in the headwater region of the HRB in the recent past (1995–2014) and near future (2015–2024). By taking advantage of an integrated modeling approach which combines the Markov chain, Dyna-CLUE and SWAT models, we specifically aim to address the following questions: (i) how has the land use and climate changed in the recent past from 1995 to 2014? (ii) what are their roles in affecting hydrological regimes? and (iii) how may the hydrological conditions be impacted by land use change and/or climate variability in the near future, from 2015 to 2024, given different scenarios?

## Materials and Methods

### 2.1 Study area

**Fig 1:**
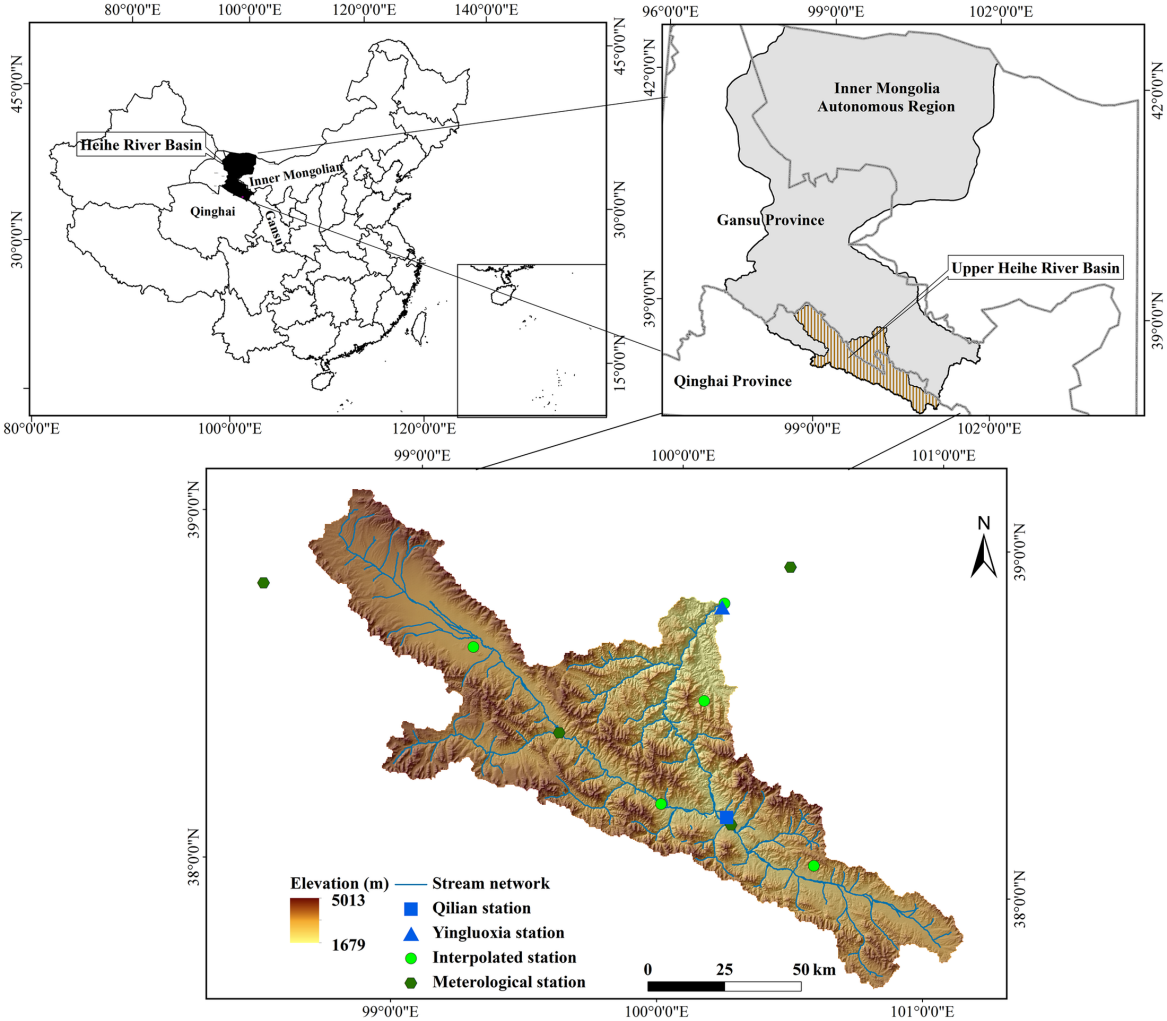
Location of the study area.

The Heihe River Basin (HRB) is located in the arid region of northwest China and lies between longitudes 98° and 101°30’ E and latitudes 38° and 42° N. The area of the HRB is approximately 128,000 sq. km with a mainstream length of 821 km. The Heihe River originates from the southern Qilian Mountains in Qinghai Province, and flows through the Hexi Corridor of Gansu Province and ends in the northern Juyan Lakes of Inner Mongolian. The headwater region (i.e., the upper HRB), which refers to the mountainous area of the Qilian Mountains extending to the Yingluoxia hydrological station (outlet of the mountain), is selected as the study area (Fig 1). The upper HRB is characterized by steep terrain, with elevation ranges from 1679 to 5013 m above sea level. In this region, the Mesozoic and Mesozoic strata, and the structural fractures are extensively developed, with widely distributed bedrock fissure water [35]. The rock types include ophiolite, blueschist, pyroclastic rock, sandstone and granite [36]. A thrust fault distributes along the foot of the Qilian Mountains in a NW–SE direction, which makes it difficult for mountain groundwater to flow out laterally and therefore most are transformed into streamflow before entering the plain oasis [37]. The upper HRB belongs to the semi-arid and sub-humid temperate continental monsoon climate zone, with an average annual precipitation of about 450 mm from 1960 to 2014, of which about 70–80% falls from June to September while less than 5% in the months from December to February [19, 38]. The mean annual temperature is lower than 2°C and the mean annual pan evaporation is approximately 700 mm [39]. There are six primary land-use types in the study area, i.e., farmland, forest, grassland, water body, built-up land and unutilized land, among which the former three occupy more than 95% of the total land area according to the local land use/cover data in the year 2011 derived from 30-m Landsat TM/ETM+ images [40]. The soil distribution in the study area exhibits distinct vertical zonality, due to the effects of mountain climate, terrain and vegetation. The predominant soil types include alpine meadow soil, alpine chestnut soil, subalpine shrub meadow soil and alpine frost desert soil [41].

### 2.2 Hydrological model

#### 2.2.1 Model description

SWAT is a physically based, computationally efficient, semi-distributed watershed model and is designed to simulate and predict the impacts of land management practices on water, sediment and agricultural chemical yields in large complex watersheds with varying soils, land use and management conditions over long periods of time [42]. The model was selected in this research because of its minimum input data and capacity to provide continuous, long-term simulations [43]. Moreover, the model has been widely used in different catchments across the world and proved to be an effective tool to examine hydrological responses to land-use and climate changes (e.g., [44], [45], [46] and [47]). In SWAT, the target watershed is divided into subbasins linked by the channel network, which are further subdivided into a number of hydrological response units (HRUs) of homogeneous land use, slope, management and soil characteristics. Taken HRU as the basic unit, hydrological components, nutrients and sediment yield are simulated and then aggregated for each subbasin. The model description is detailed at http://swat.tamu.edu/.

#### 2.2.2 Model setup

For this study, the model was set up using the GIS interface of SWAT (ArcSWAT 2009.93.7b). The basic SWAT inputs include digital elevation model (DEM), land use map, soil information and meteorological data. The 90-m local DEM that clipped from the ASTER Global DEM and the 2000 land use map at a scale of 1: 100,000 were collected from the Science Data Center for Cold and Arid Regions (http://westdc.westgis.ac.cn). The 1: 1000,000 soil map was taken from the second State Soil Survey of China (i.e., the latest nation-wide soil survey in China) completed in the early 1980s. Further data on soil physical properties such as texture, soil depth, available water capacity, saturated hydraulic conductivity, bulk density, organic content, clay content and silt content were partly obtained from Gansu Soil Handbook [48] and partly from the field observations as described in Yu et al. (2013) [41] and Lai et al. (2013) [49]. The meteorological dataset for the period 1991–2014 were collected from the China Meteorological Data Sharing Service System (http://cdc.nmic.cn/), including daily data of maximum and minimum temperature, precipitation, relative humidity and wind speed measured at the Qilian, Yeniugou, Tuole and Zhangye stations (Fig 1). The few precipitation stations within the upper HRB may lead to insufficiency in capturing spatial variability of precipitation. Thus, we selected five extra locations as the interpolated stations (Fig 1), and interpolated their precipitation time series with a tool called meteorological distribution system for high-resolution terrestrial modeling (MicroMet) [50]. The SWAT model performance can be improved by adding those interpolated precipitation stations.

The studied watershed was divided into 24 subbasins with a threshold area of 20,000 ha. The subbasins were further discretized to 143 HRUs by setting land use and soil threshold levels to 5% and 10%, respectively. The single slope discretization option was selected in the process of creation of HRUs. The agricultural management was parameterized using the SWAT default values, owing to a very small proportion of agricultural lands (<1%) in the watershed. In addition, the Penman–Monteith method was chosen to estimate potential evapotranspiration, the soil conservation service curve number (SCS-CN) method to compute surface runoff and the variable storage method to route channel flow.

The SWAT model was calibrated using monthly streamflow data for a period of eight years (1994–2001) at the Yingluoxia station which is the outlet of the upper HRB. The calibration was performed through a “trial and error” process by manually adjusting the sensitive parameters recommended in the published literatures [30, 41, 49, 51]. The model was warmed-up for 3 years (1991–1993) to mitigate the effects of inaccurate initial conditions. After calibration, nine-year observations (2002–2009) at the Yingluoxia station and additional nine-year records (2000–2008) at the Qilian station which is the outlet of a sub-basin within the upper HRB, known as the Babao river basin (Fig 1), were used for model validation. The performance of SWAT model during both calibration and validation periods was assessed using three qualitative statistics recommended by Moriasi et al. (2007) [52], i.e., (i) Nash-Sutcliffe efficiency (NSE) [53], (ii) percent bias (PBIAS), and (iii) RMSE-observations standard deviation ratio (RSR).

### 2.3 Land use models

In this study, two land use models, namely the Markov chain model and the Dynamic Conversion of Land-Use and its Effects (Dyna-CLUE) model, were combined to project land use changes in the upper HRB from 2012 to 2024. The Markov chain determines the temporal change of each land use type based on transition probability matrix while Dyna-CLUE controls the spatial pattern changes based on empirically quantified relations between land use and its driving factors in combination with dynamic modeling of competition between land use types.

#### 2.3.1 Markov chain

As the simplest Markov model, Markov chain describes a discrete stochastic process with the “memorylessness” (also called Markov property), i.e., the next state of a process depends on the current state and not on the sequence of states that preceded it [54]. The model has been proven to be a suitable and convenient tool for projecting the changes of land use structure in a certain region because: (i) different land use types may be converted into each other [55]; (ii) mutual conversions between land-use types are difficult to describe precisely with some special functions [56]; and (iii) the land-use status are relatively stable over a short period [57]. In the Markov chain model, the change of land use structure is projected using Eqs 1 and 2.

$${\text{S}}_{t+1}={\text{S}}_{t}\times P_{ij}$$ (1)

$$P_{ij}=\left[\begin{array}{ccc} P_{11} & \begin{array}{cc} P_{12} & \ldots \end{array} & P_{1n}\\ P_{21} & \begin{array}{cc} P_{22} & \ldots \end{array} & P_{2n}\\ \begin{array}{c} \vdots \\ P_{n1} \end{array} & \begin{array}{cc} \begin{array}{c} \vdots \\ n2 \end{array} & \begin{array}{c} \vdots \\ \ldots \end{array} \end{array} & \begin{array}{c} \vdots \\ P_{nn} \end{array} \end{array}\right]$$ (2)

where *S*_*t*+1_ and *S*_*t*_ are the land use structure at time *t+*1 and *t*; *P*_*ij*_ is the transformation probability of the land use type *i* into the type *j* from prophase to telophase; *n* is the number of land use types in the study area.

#### 2.3.2 Dyna-CLUE

Dyna-CLUE is an enhanced empirical land use model which can simulate multiple land-use types simultaneously through the combination of the top-down allocation of land use change to grid cells with a bottom-up determination of conversions for specific land use transitions [58, 59]. The model was selected because it is freely available and has a friendly user interface, and more importantly, has been widely reported to be a reliable tool to project land use changes and to assess potential hydrological impacts together with hydrological models [60–63]. The model mainly includes two modules: a non-spatial demand module and a spatially explicit allocation module. The non-spatial module, which is exogenous to the Dyna-CLUE modeling framework, determines the area changes of land use types at the aggregate level. It manages to answer the question “At what rates are land-use changes likely to progress?”. In this study, the land-use area changes in the upper HRB from 2012 to 2024 were projected using the Markov chain based on the transition probability matrix from 2000 to 2011. The allocation module, on the other hand, translates the demands into land use changes at different locations within the same time frame. Thus, it manages to address another question “Where are land-use changes likely to take place?”.

For each location (grid cell) at a time, the most preferred land use type is derived from a combination of the location suitability, neighborhood suitability, conversion elasticity and competitive advantage [58]. Moreover, user-specified spatial restrictions and conversion matrix constrain specific land use conversion in a target region. The location suitability and neighborhood suitability can be empirically estimated by logistic regression based on the relations between land use and its driving factors, and between land use and its enrichment factors, respectively [59, 64]. In this study, the logistic regression was performed using the land use map for the year 2000 and twelve selected land use drivers, i.e., (i) elevation, (ii) slope, (iii) aspect, (iv) soil type, (v) distances to nearest road, (vi) to nearest railway, (vii) to nearest river, (viii) to nearest reservoir, and (ix) to nearest residential area, (x) GDP per land area, (xi) GDP per capita and (xii) population density.

The land-use data and driving factors used in this study were collected from the Science Data Center for Cold and Arid Regions and the Statistical Yearbooks of Gansu and Qinghai Provinces. The land use/cover data in 1986 were obtained from the National Land Use/cover Database of China (NLUD-C) at a scale of 1:100,000 [65]. The land use/cover datasets in 2000 and 2011 were derived from the Landsat TM/ETM+ satellite images through visual interpretation and have been verified to have a very high classification accuracy [26, 40, 66]. The land-use types in each dataset were reclassified into six primary types: farmland, forest, grassland, water body, built-up land and unutilized land.

In order to perform valid projection of future land use patterns, it is essential to validate the applicability of land use models. In this study, the 1986 and 2000 land use maps were utilized as the base maps to develop and calibrate the model, whereas the 2011 map was used for validation. The area demands for all land use types from 2000 to 2011 were first calculated using the Markov chain based on the transition probability matrix from 1986 to 2000. The land-use patterns from 2000 to 2011 were then simulated using the Dyna-CLUE model. The model accuracy was assessed by comparing the projected and the real 2011 land-use patterns using overall accuracy, Kappa coefficient and producer’s accuracy [67]. Higher values of the first two indexes indicate higher agreement between the actual and the simulated maps. Likewise, larger producer’s accuracy indicates greater similarity between the pair of classes tested.

### 2.4 Climate change scenarios

The GCM projection and hypothetical approach are two primary methods to generate future climate change scenarios for climate change impact assessment. The physically based GCMs act as one of the major tools to study the nature of climate change [68]. However, despite the continual development, the significant mismatches between GCMs and hydrological models in terms of time and space still exist and bring about vital challenges for the local or regional assessment of hydrological impacts. Albeit many downscaling techniques, either dynamical or empirical, have been developed to narrow the gap, they frequently bring more errors and uncertainties to the results [1]. Furthermore, GCMs are more capable of simulating the free troposphere climate than the surface climate and usually attached with much uncertainty on the magnitude of the changes in temperature and precipitation, which are, however, the primary controlling factors for watershed-scale hydrological processes [16, 68, 69]. Given the deficiencies of GCM projections, hypothetical scenarios are preferred to some extent to investigate watershed hydrological responses [15, 70–72].

Hypothetical scenarios are purposively designed to represent changes in climate variables, such as temperature and precipitation. The variables can be specified according to the analyses of long-term historical observations or a qualitative interpretation of GCM predictions [14, 73]. Generally, the generation of hypothetical scenarios consists of two steps [74]. The average annual changes in precipitation and temperature for a fixed time-slice are estimated firstly (typically, let ΔT denote absolute change (°C) and ΔP relative change (%)). Then, the historic temperature and precipitation series with the same length as the fixed time-slice are perturbed by adding ΔT and multiplying (1 + ΔP), respectively.

**Fig 2:**
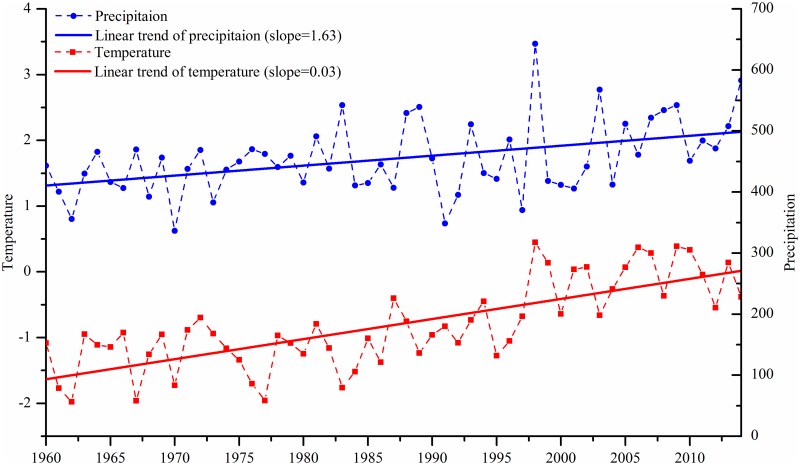
Trends of temperature and precipitation in the headwater region of the HRB during 1960–2014.

To select a reasonable range for the climate change scenarios, the variations of temperature and precipitation from 1960 to 2014 were analyzed, as shown in Fig 2. It is obvious that both temperature and precipitation show an increasing trend, indicating that the climate is becoming warmer and wetter in the study area, in line with the common trend in the arid region of northwest China [13] and the GCM projections in the HRB [30, 75]. The increase in precipitation is intimately bound up with the northward migration of the Eastern Asian summer monsoon (EASM) which is induced by the expansion and enhancement of the western Pacific subtropical high (WPSH) in the context of global warming [75]. A 20-year moving window method is used to calculate the largest and median increments of mean annual temperature and precipitation for two neighboring time slices (10 years) over the period 1960–2014, which are 0.89°C and 10.72%, and 0.41°C and 5.00%, respectively. Then, four hypothetical climate scenarios (Table 2) were established, through perturbing the base series of temperature and precipitation (2005–2014), to represent a range of possible climate conditions in the near future (2015–2024). Other climatic variables such as relative humidity, wind speed and solar radiation were simply considered to be unchanged for each scenario.

**Table 2:** Hypothetical climate change scenarios.

Scenario	Wettest and Warmest	Wettest and warm	Wet and Warmest	Wet and Warm
**ΔT (°C)**	0.89	0.41	0.89	0.41
**ΔP (%)**	10.72	10.72	5.00	5.00

### 2.5 Hydrological impact assessment

#### 2.5.1 Impacts of land use and climate changes in the past

The hydrological impacts of changes in land use and climate in the recent past (1995–2014) were investigated by the approach of “one factor at a time”, which allows changing one factor at a time while fixing other conditions [76]. Meteorological data from 1995 to 2014 were selected and split into two periods, i.e., 1995–2004 and 2005–2014, during which the land use pattern was represented by the land use maps in 2000 and 2011, respectively. Through the combination of two periods of climatic data and two land use maps, four modeling experiments (Table 3) were designed to quantify the contributions of land use and climate changes to hydrological variations in the recent past.

**Table 3:** Model experiments for assessing hydrological impacts of land use and climate changes in the past.

Model experiment	Land use pattern	Climatic conditions
**E1**	2000	1995–2004
**E2**	2011	1995–2004
**E3**	2000	2005–2014
**E4**	2011	2005–2014

#### 2.5.2 Potential impacts of future land use and climate changes

The projected land use pattern in 2024 and the hypothetical climate scenarios for the period 2015–2024 were incorporated into the calibrated SWAT model to assess potential impacts of future land use changes and climate variabilities on hydrology regimes. Both the separate and joint effects of land use change and climate variability were evaluated with the following scenarios.

1. *Baseline*: Most recent land use pattern for the year 2011 and climatic conditions from 2005 to 2014;
2. *Land use change only (LUCC)*: Projected land use pattern in 2024 and climate conditions from 2005 to 2014;
3. *Climate change only (CC)*: Land use pattern for the year 2011 and climatic conditions from 2015 to 2024 under four hypothetical climate scenarios (Wettest and Warmest, Wettest and Warm, Wet and Warmest, Wet and Warm);
4. *Land use and climate changes (LUCC+CC)*: Projected land use pattern in 2024 and climatic conditions from 2015 to 2024 under four hypothetical climate scenarios.

Under each scenario, besides the trend and magnitude of the variations of hydrological components (surface runoff, groundwater discharge, ET and streamflow), the statistical significances of these changes were also evaluated by performing the two tailed paired t-test. The target level of significance was set to 0.05 and 0.1.

## Results and Discussion

### 3.1 Model calibration and validation

#### 3.1.1 Validation of the combined land use models

Based on the transition probability matrix from 1986 to 2000, the combined Markov chain and Dyna-CLUE models were performed to simulate land use changes from 2001 to 2011. The simulated and actual land use patterns for the year 2011 were compared; the resultant Kappa coefficient and overall accuracy are 0.87 and 0.94, respectively, indicating a reliable land use modeling [61]. All land use types except for forest have high producer’s accuracy within the study area (Table 4). The relative low accuracy in modeling forest is because of overestimated deforestation rate from 2001 to 2011 in comparison with the reference period from 1986 to 2000, which is highly related to the ecological rehabilitation projects implemented in the HRB [77, 78]. Overall, the combined models were proven to be usable for projecting future land use patterns in the study area.

**Table 4:** Validation metrics for the combined land use models.

Overall accuracy	Kappa coefficient	Producer’s accuracy					
		farmland	Forest	Grassland	Water body	Built-up land	Unutilized land
0.94	0.87	0.98	0.69	0.96	0.88	0.84	0.92

#### 3.1.2 Calibration and validation of SWAT

The calibrated parameters including their description, initial and optimal values are presented in Table 5. The soil related sensitive parameters such as saturated hydrological conductivity (SOL_K) and soil available water capacity (SOL_AWC) were not calibrated because they were obtained through field observations and treated to be “true” [41, 49]. The soil evaporation compensation factor (ESCO) allows users to specify the soil evaporation demand distribution with depth, accounting for the impacts of capillary action, crusting and cracks. The relatively high ESCO (0.925) indicates that the soil evaporation probably plays a weaker role in the whole evaporation process, possibly owing to the high vegetation coverage and low air temperature in the upper HRB [79]. The baseflow recession constant (ALPHA_BF), a direct index of groundwater flow response to changes in recharge, was determined mainly by referring to the works of Huang et al. (2004) [80] and Gan et al. (2015) [81], in which the ALPHA_BF was estimated using the digital filter and historical streamflow records. The ALPHA_BF of 0.002 was very small, indicating slow drainage and large storage in the shallow aquifer of the study basin. The slow drainage of shallow groundwater flow, which is possibly related to the complex geological structures of the upper HRB such as widely distributed faults [82], was reported by Wang et al. (2009) [83] who investigated the runoff characteristics of the Heihe River using stable isotope technique. The large shallow groundwater storage is consistent with the hydrogeological survey results in the HRB [82]. The parameter GW_DELAY is the lag between the time that water exits soil profile and enters the shallow aquifer. Its lower value (10 days) may be explained by higher water content in the vadose zone, as a result of substantial replenishment from precipitation and snowmelt, which makes it easy for water to reach the groundwater zone [84]. Another parameter REVAPMN that is intimately correlated with groundwater flow was calibrated to 500 mm by referring to the study of Zhang et al. (2014) [85], and presented good fit for the low flows. The precipitation and temperature lapse ratios (PLAPS and TLAPS) were eventually set to 82 mm/km and -5°C/km, respectively, following the study of Li (2012) [86] in which they were estimated using ten-year (2000–2009) station records. Considering the study basin located in an alpine region, the two parameters (SMFMN and SMTMP) associated with snow melting processes were adjusted to 3.5 mm H_2_O/°C-day and -1°C, respectively, on a trial-and-error basis.

**Table 5:** Sensitive parameters and their initial and optimal values.

Parameter	Description	Initial value	Optimal value
ESCO	Soil evaporation compensation factor	0.95	0.925
ALPHA_BF	Baseflow recession constant	0.048	0.002
GW_DELAY	Groundwater delay time	31	10
REVAPMN	Threshold water level in shallow aquifer for “revap”	1.0	500
TLAPS	Temperature lapse rate	0.0	-5.0
PLAPS	Precipitation lapse rate	0.0	82.0
SMFMN	Melt factor for snow on December 21	4.5	3.5
SMTMP	Snowmelt base temperature	0.5	-1.0

**Fig 3:**
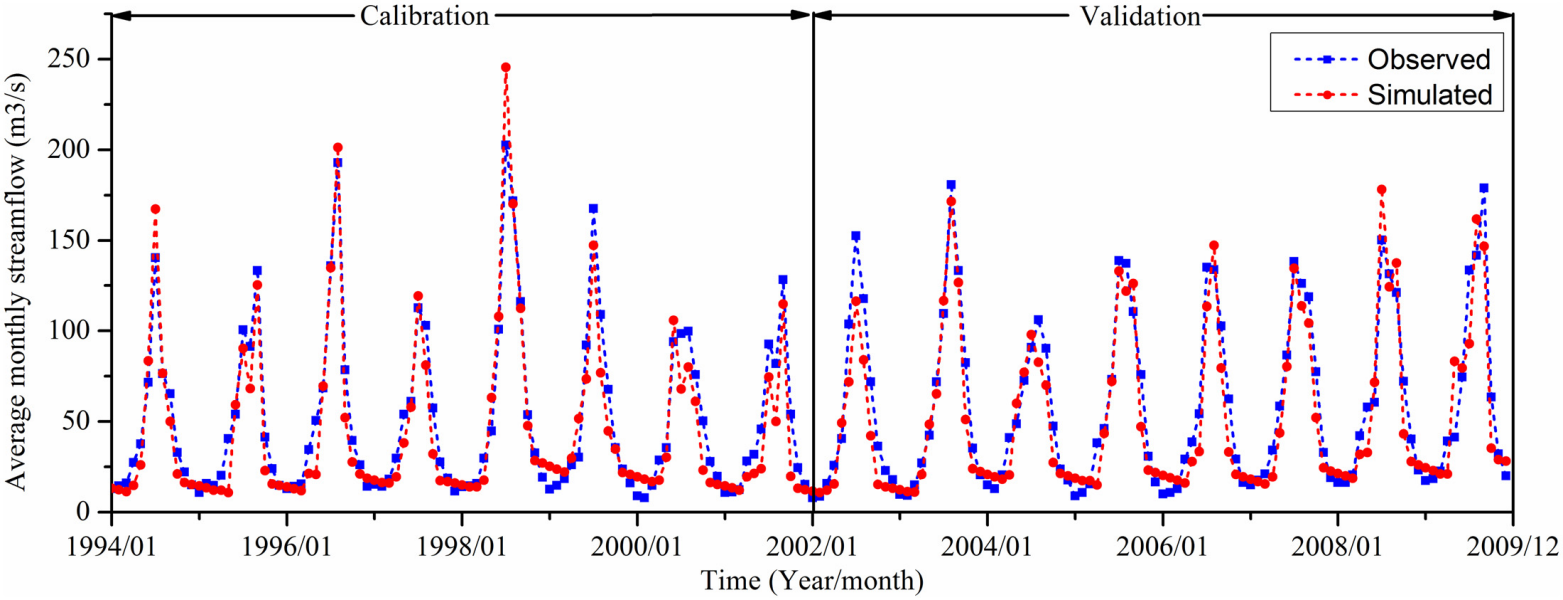
Observed and simulated streamflow at the Yingluoxia station for the calibration (1994–2001) and validation (2002–2009) periods.

As shown in Fig 3, the simulated average monthly streamflow agrees well with the observed records at the outlet of the upper HRB, i.e., the Yingluoxia station. The model efficiency statistics during the calibration and validation periods are given in Table 6. NSE, PBIAS and RSR are 0.898, 10.5% and 0.320, respectively, for the calibration period, and 0.881, 10.1% and 0.345, respectively, for the validation period. Validation at the Qilian station also indicates a good performance of the model. The SWAT simulation tends to underestimate flows at both stations. This may be related to the changes in land use and the model deficiency in capturing some hydrological processes such as snowmelt and storm runoff in the mountainous area [20, 41]. Regardless, the calibration and validation results indicate good streamflow simulations according to the model evaluation guidelines proposed by Moriasi et al. (2007) [52].

**Table 6:** Efficiency metrics for monthly streamflow simulation at the Yingluoxia station during the calibration and validation periods.

Hydrological Stations	Period	Efficiency metrics		
		NSE	PBIAS	RSR
Yingluoxia	Calibration (1994–2001)	0.898	10.5%	0.320
	Validation (2002–2009)	0.881	10.1%	0.345
Qilian	Validation (2002–2008)	0.724	6.6%	0.489

In this study, the actual ET was not used to calibrate and validate the model due to a lack of observed data. As the sum of streamflow and ET in a year is almost equal to the total precipitation for a long time period [23], a good streamflow simulation also implies a good ET simulation. Moreover, the simulated ET is comparable with other studies. Cheng et al. (2014) [87] estimated the average annual ET from remote sensing data for the upper HRB over 2001–2012, which is about 363 mm, a little higher than our estimation of 323 mm. Zang et al. (2012) [88] and Wu et al. (2014) [51] obtained the ET distribution over the upper HRB using the SWAT model, which is in similar magnitude with our results. In the upper HRB, precipitation is scarce in the period from November to March during which the streamflow is mainly recharged by groundwater. The agreement between the simulated and observed streamflow during this period (Fig 3), to some degree, implies a good simulation of groundwater discharge. Moreover, the baseflow index (the ratio of groundwater discharge to streamflow) was estimated to be about 0.40 in the upper HRB through baseflow separation using various methods such as digital filter and graphical method [89], which is a little higher than our simulation result (0.35). The discrepancy is acceptable because all those separation methods are subject to uncertainties [90], and moreover, the model structure of SWAT may not able to fully capture the characteristics of groundwater flow. Although the proportions of surface flow and lateral flow are hard to determine, the lateral flow is believed to contribute more to streamflow than surface runoff due to steep terrain and loose soil with large porosity in the study area [51], which aligns well with our simulation.

Overall, it can be concluded that the calibrated SWAT model is applicable to the study area and can be applied to evaluate the impacts of land use change and climate variability on hydrological components.

### 3.2 Land use changes

**Fig 4:**
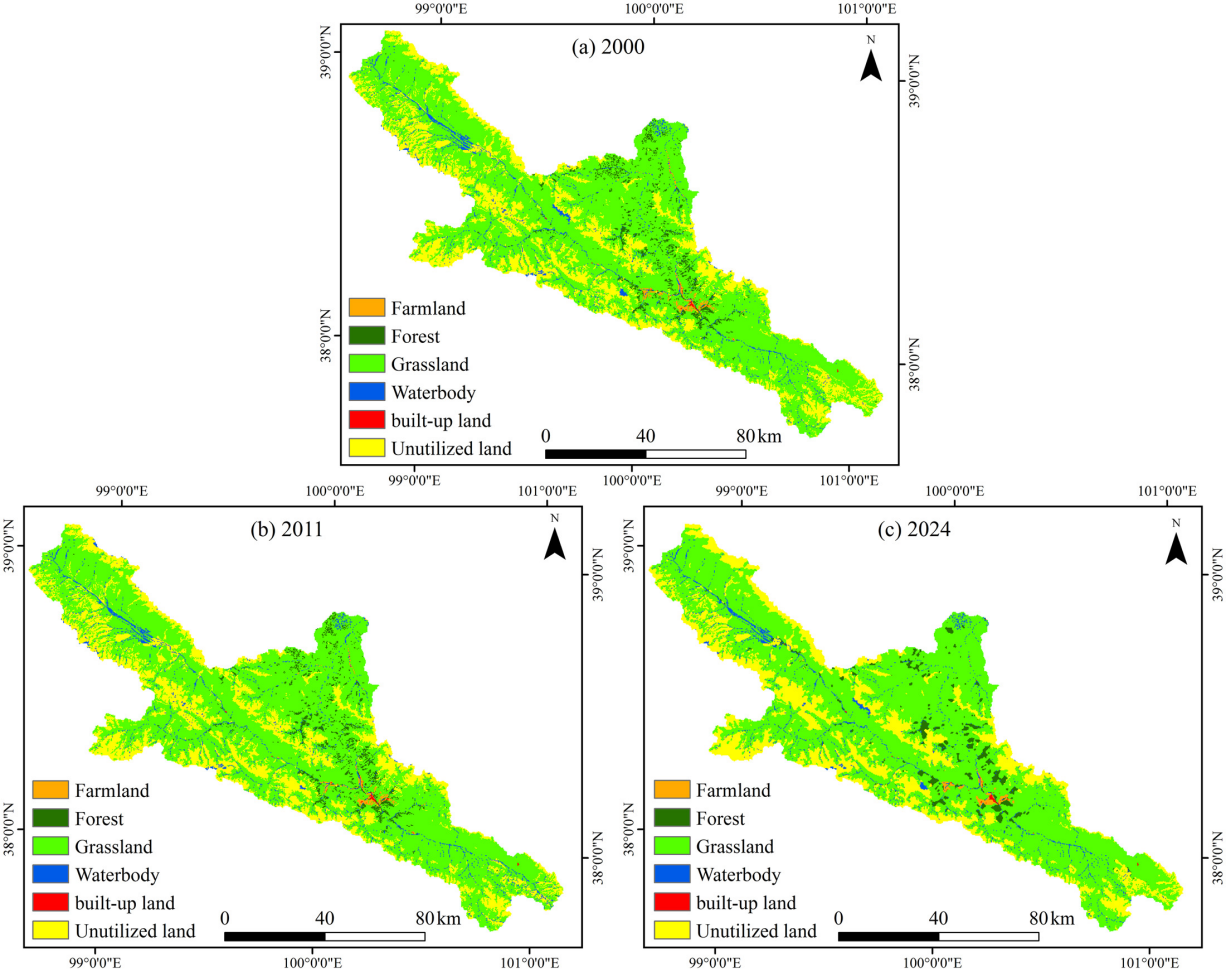
Actual land use maps for the years 2000 (a) and 2011 (b) and the projected map for the year 2024 (c) in the upper HRB.

Land use changes in the periods of 2000–2011 and 2011–2024 are summarized in Table 7 and illustrated in Fig 4. The land use patterns in 2000 and 2011 (Fig 4a and 4b) are actual while the pattern in 2024 (Fig 4c) was projected by the combined land use models based on the transition probability matrix of land use changes from 2000 to 2011. It is clear that grassland and unutilized land are the largest and most widely distributed land-use types in the study area. The main variation trend from 2000 to 2011 is the decrease in farmland and the increase in grassland. The area of farmland declines from 6,219 ha to 5,157 ha, a decrease of 1,062 ha or 17.08% of the total farmland area in 2000. By contrast, the area of grassland increases from 67,4343 ha to 675,459 ha, an increase of 1,116 ha or 0.17% of the total grassland area in 2000. There are no pronounced trends over this period for other land use types, i.e., forest, unutilized land, water body and built-up land. The land use projection indicates that there will be a continuous decrease in farmland area and, meanwhile, a constant increase in grassland area (Fig 4).

**Table 7:** Changes of land use in the upper HRB from 2000 to 2024.

Class	2000		2011		2024		2000–2011		2011–2024	
	Area (ha)	Percent (%)	Area (ha)	Percent (%)	Area (ha)	Percent (%)	Area change (ha)	Percent (%)	Area change (ha)	Percent (%)
**Farmland**	6,219	0.64	5,157	0.53	4,536	0.53	-1,062	-17.08	-621	-12.04
**Forest**	26,703	2.74	26,568	2.72	26,451	2.72	-135	-0.51	-117	-0.44
**Grassland**	674,343	69.10	675,459	69.21	677,826	69.21	1,116	0.17	2,367	0.35
**Water body**	30,978	3.17	31,617	3.24	29,511	3.24	639	2.06	-2,106	-6.66
**Built-up land**	738	0.08	873	0.09	945	0.09	135	18.29	72	8.24
**Unutilized land**	236,934	24.28	236,241	24.21	236,646	24.21	-693	-0.29	405	0.17

The transition matrix of land use change from 2000 to 2011 is presented in Table 8. The results indicate that the conversion to grassland mainly contributes to the decrease in farmland. The increase in grassland is primarily attributed to the transition from unutilized land, forest land and farmland. The most prominent land use changes, i.e., the increase in grassland and the decline in farmland, are intimately related with the implementation of the Grain for Green project (GGP), which was initiated by the Chinese government in 1999 and was designed to convert sloping farmland and wasteland back to forest and grassland in order to halt soil erosion [91]. Besides, the regional ecological rehabilitation projects such as the Ecological Water Transfer and Rehabilitation Project (EWTRP) [78], the Ecological Water Diversion Project (EWDP) [77] and the recent Management Planning of Heihe River Basin [92], are also favorable to ecological land development in the upper HRB, although they were mainly implemented in the middle oasis areas. In spite of economic development and urban expansion, the growth in built-up land is small, mostly at the expense of farmland. The spatial extent of water body increases, primarily because of the conversion of unutilized land, which is possibly related to the wetter climate in the headwater region of the HRB in recent years.

**Table 8:** Transition matrix of land use change from 2000 to 2011 (ha).

2000	2011					
	Farmland	Forest	Grassland	Water body	Built-up land	Unutilized land
**Farmland**	5,076	63	963	18	99	0
**Forest**	9	24,228	2,448	9	0	9
**Grassland**	27	1,980	661,797	297	45	10,197
**Water body**	27	153	315	28,935	0	1,548
**Built-up land**	0	0	9	0	729	0
**Unutilized land**	18	144	9,927	2,358	0	224,487

### 3.3 Effects of past land use change and climate variability

Table 9 presents the mean annual hydrological components (specifically surface runoff, groundwater discharge, evapotranspiration (ET) and streamflow) experimentally simulated by SWAT with varying land use patterns and climatic conditions. The differences between experiments E1 and E2 imply the sole effects of land use change. Compared with E1, surface runoff, groundwater discharge and streamflow in E2 are going down, while ET is going up. The variations can be explained by the decrease in the extension of farmland and unutilized land, both of which have low ET capacities and high water yield capacities, and the expansion of grassland, which conversely has a high ET capacity and a low water yield capacity [28, 93, 94]. The obvious distinctions between E1 and E3 on the other side indicate the effects solely induced by climate variability. The climate variations cause remarkable increases in surface runoff, groundwater discharge, ET and streamflow by 19.16%, 11.93%, 9.33% and 12.24%, respectively, as a result of prominent increase in precipitation (+51.75 mm or +11.37%).

**Table 9:** Mean annual hydrological components in different model experiments with different land use patterns and climate conditions (values in parentheses indicate percentages (%) of change relative to E1).

Experiment	Land use pattern	Climatic conditions	Precipitation (mm/year)	Surface runoff (mm/year)	Groundwater discharge (mm/year)	ET (mm/year)	Streamflow (mm/year)
**E1**	2000	1995–2004	455.21	33.62	52.23	302.57	151.54
**E2**	2011	1995–2004	455.21 (0)	33.32 (-0.89)	51.95 (-0.54)	303.07 (0.17)	151.07 (-0.31)
**E3**	2000	2005–2014	506.96 (11.37)	40.06 (19.16)	58.46 (11.93)	330.79 (9.33)	170.09 (12.24)
**E4**	2011	2005–2014	506.96 (11.37)	39.77 (18.29)	58.25 (11.53)	331.21 (9.47)	169.66 (11.96)

The contrasts between E1 and E4 imply the combined hydrological impacts of both land use change and climate variability. The results show that the joint effects lead to consistent growths in surface runoff, groundwater discharge, ET and streamflow by 18.29%, 11.53%, 9.47% and 11.96%, respectively, which are highly similar to those solely induced by climate changes. It suggests that climate change plays a more pronounced role than land use change in impacting hydrology regimes during the recent past from 1995 to 2014. The combined effects of land use change and climate variability are not a simple addition of the individual impacts owing to the interactions between land use change and climate variability represented in the SWAT model [3, 76]. For example, as presented in Table 9, the streamflow is altered by -0.31% and +12.24% as a separate result of land use changes (E2) and climate changes (E3), whereas changed by +11.96%, as a result of joint effects, which is 0.03% higher than the sum of -0.31% and +12.24%.

**Fig 5:**
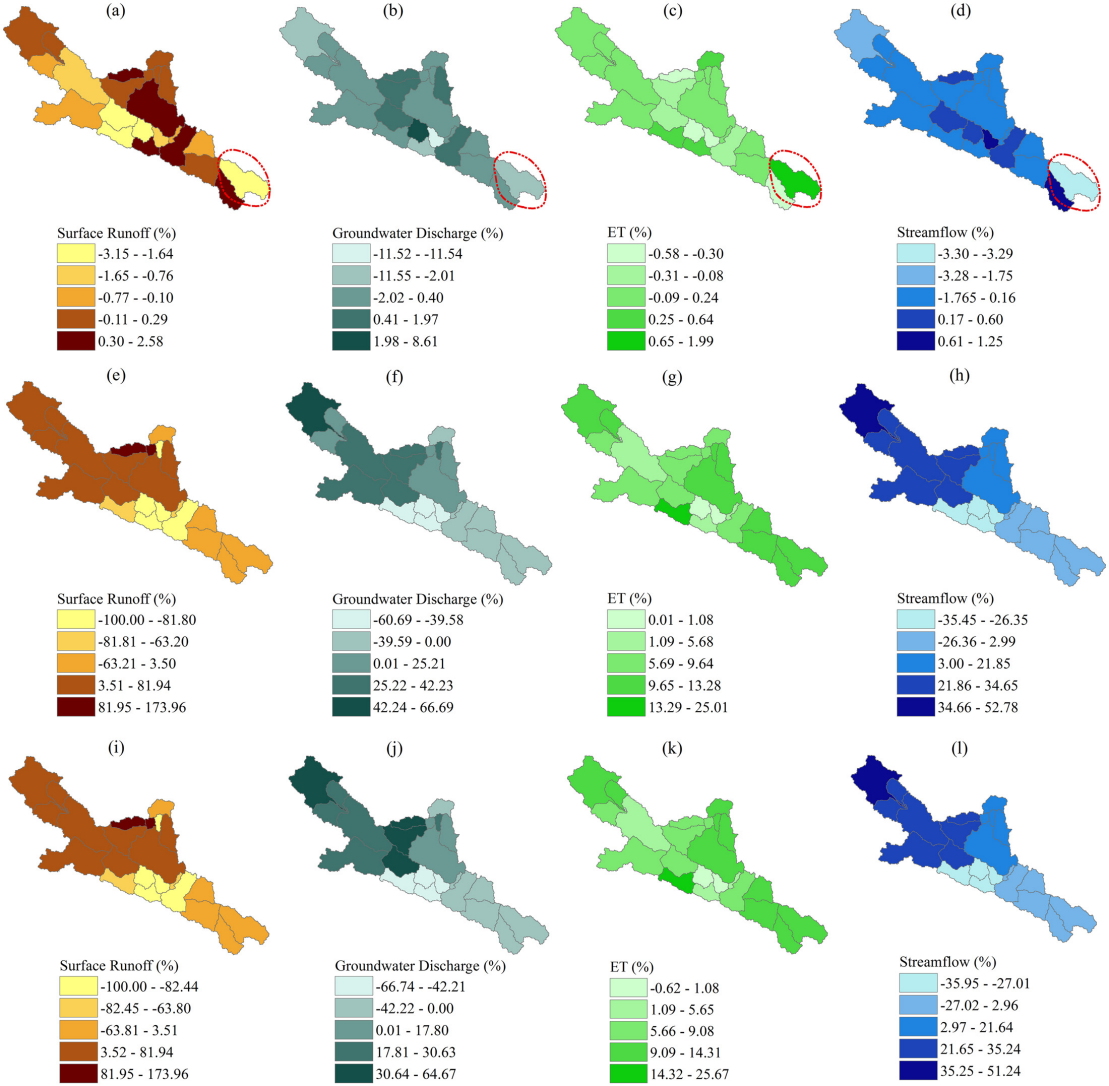
Spatial pattern of the changes in surface runoff, groundwater discharge, ET and streamflow. The top panel (a, b, c and d) shows the changes ((E2-E1)/E1×100) induced by land use change. The middle panel (e, f, g and h) shows the changes ((E3-E1)/E1×100) induced by climate variability. The bottom panel (i, j, k and l) shows the changes ((E4-E1)/E1×100) induced by the combined land use and climate changes.

Fig 5 shows the changes in mean annual hydrological components at sub-basin levels. The top panel of (Fig 5a, 5b, 5c and 5d) illustrates the hydrological effects solely induced by land use changes. The spatial variability of the changes in each hydrological component is apparent, due to the varying land use changes among different subbasins (Fig 4). Taken the sub-basin marked by an ellipse as an example (Fig 5), it undergoes more pronounced hydrological variations than the rest. This is because this sub-basin undergoes more significant changes in land use, particularly the conversion from unused land to grassland. On sub-basin scales, the percentage changes in surface runoff, groundwater discharge, ET and streamflow caused by land use changes range from -3.15% to 2.58%, from -11.52% to 8.61%, from -0.58% to 1.99% and from -3.30% to 1.25%, respectively. The middle panel of (Fig 5e, 5f, 5g and 5h) shows the effects of climate changes, which exhibit an obvious spatial difference as well. The changes of surface runoff, groundwater discharge and streamflow tend to be positive in the northwestern part and negative in the southeastern part. The ET is observed to increase in all sub-basins, but with varying magnitudes. The bottom panel of (Fig 5e, 5f, 5g and 5h) depicts the spatial patterns of change in hydrological components jointly induced by land use and climate changes, which appear to be highly similar to those solely induced by climate changes.

### 3.4 Combined effects of future land use and climate changes

The simulated mean annual hydrological components under different scenarios, i.e., the baseline, land use change only (LUCC), climate change only (CC), and land use and climate changes (LUCC+CC), are presented in Table 10. In comparison to the baseline scenario, an increase in ET (0.20%) and reductions in surface runoff (0.15%), groundwater discharge (1.02%) and streamflow (0.27%) are observed under the LUCC scenario. The variations can be attributed to the fact, according to the projection, there will be more grassland and less farmland in the headwater region in 2024 than 2011. The influences of land use changes over hydrological regimes in the near future seem to be slight, similar to those in the recent past. It could be argued that the basin-wide hydrological impacts are actually averaged over the entire basin, which means the positive and negative effects of land use changes occurred in different subbasins can offset each other [29, 76, 95]. Moreover, the absolute amount of changed land use area is relatively small in comparison with the size of the whole study area, resulting in small aggregated hydrological impacts. The overall impacts of a studied basin would become more pronounced if the land use changes are more extensive, as showcased by the sub-basin marked by the ellipse (Fig 5) in which the land use changes are pronounced and the hydrological impacts are consequently prominent.

**Table 10:** Mean annual hydrological components for different future scenarios (values in parentheses indicate percentages (%) of change relative to baseline scenario).

Scenario	Precipitation (mm/year)	Surface Runoff (mm/year)	Groundwater Discharge (mm/year)	ET (mm/year)	Streamflow (mm/year)
**Baseline**	506.96	39.77	58.25	331.21	169.66
**Land use change only (LUCC)**	506.96 (0)	39.71 (-0.15)	57.66 (-1.02)	331.88 (0.2)	169.20 (-0.27)
**Climate change only (CC)**					
**Wettest and Warmest**	561.14 (10.69)	51.27 (28.91)	67.78 (16.35)	351.84 (6.23)	201.17 (18.57)
**Wettest and Warm**	561.14 (10.69)	54.13 (36.1)	71.79 (23.25)	344.28 (3.95)	208.13 (22.67)
**Wet and Warmest**	531.95 (4.93)	42.55 (6.98)	59.19 (1.61)	347.36 (4.88)	178.43 (5.17)
**Wet and Warm**	531.95 (4.93)	45.07 (13.33)	62.87 (7.94)	340.70 (2.86)	184.67 (8.85)
**Land use and climate changes (LUCC+CC)**					
**Wettest and Warmest**	561.14 (10.69)	51.18 (28.7)	67.05 (15.11)	352.50 (6.43)	200.57 (18.22)
**Wettest and Warm**	561.14 (10.69)	54.03 (35.84)	71.10 (22.07)	344.89 (4.13)	207.57 (22.34)
**Wet and Warmest**	531.95 (4.93)	42.48 (6.81)	58.50 (0.43)	347.99 (5.07)	177.86 (4.83)
**Wet and Warm**	531.95 (4.93)	44.99 (13.14)	62.21 (6.8)	341.30 (3.04)	184.11 (8.52)

The basin-wide hydrological impacts are pronounced under the CC scenarios with four different hypothetical climatic settings, i.e., Wettest and Warmest, Wettest and Warm, Wet and Warmest, and Wet and Warm. These hypothetical climate scenarios consistently result in an increase in each hydrological component, mainly owing to the increase of precipitation. The largest increases in surface runoff (36.10%), groundwater discharge (23.25%) and streamflow (22.67%) are induced by the Wettest and Warm scenario, followed by Wettest and Warmest, Wet and Warm, and Wet and Warmest. However, the largest rise in ET (6.23%) is produced by the Wettest and Warmest scenario, followed by Wet and Warmest, Wettest and Warm, and Wet and Warm. Considering the two wettest conditions, i.e., Wettest and Warmest and Wettest and Warm, under which the temperature setting in the former is 0.48°C higher than the latter, ET is simulated to be higher by 7.56 mm (2.20%), and conversely, surface runoff, groundwater discharge and streamflow are simulated to be lower by 2.86 mm (5.28%), 4.01 (5.59%) and 6.96 mm (3.34%), respectively. Similar trends can be observed under the two wet conditions. The inter-comparisons show the temperature is in negative relation to surface runoff, groundwater discharge and streamflow while precipitation is in positive relation.

In the case of LUCC+CC, the impacts on hydrological components are pronounced as well. All the components are simulated to increase under any of the four combined settings. The LUCC & Wettest and Warmest scenario project increases in surface runoff, groundwater discharge and streamflow by 28.70%, 15.11% and 18.22%, respectively, which are slightly less than the increases (28.91%, 16.35% and 18.57%) under the corresponding CC scenario (Wettest and Warmest). Similar findings can be reached for other combined LUCC+CC scenarios. It can be attributed to the tradeoff between land use change and climate variability. The land use changes offset the impacts of climate change on surface runoff, groundwater discharge and streamflow through intensifying ET. However, it is obvious that the joint effects are highly consistent with the effects of climate change. Hence, the climate change is expected to have more prominent hydrological effects than the land use change in the near future, in alignment with what have been concluded for the recent past. This finding is also consistent with the results documented in other studies performed in the HRB [25, 30] as well as other river basins (e.g., [74], [75] and [5]). If the climate in target region follows a historical trend and becomes wetter in the future, as we assumed in this study, the impacts of land use change seem to be not important. However, in case the climate becomes drier in the future, the potential impacts of land use change should not be overlooked, as the land use changes will intensify ET and consequently exacerbate the aridity condition.

By comparing Tables 9 and 10, it can be found that, in the recent past, the effects of LUCC are more pronounced on surface runoff and groundwater discharge than on streamflow and ET, while in the near future, the effects are differently more pronounced on streamflow and groundwater discharge than on surface runoff and ET. Likewise, the impacts of climate change are more prominent on surface runoff and groundwater discharge than on streamflow and ET under the Wettest and Warm scenario, whereas they are more prominent on surface runoff and streamflow than on groundwater discharge and ET under the other CC scenarios. The magnitudes of hydrological responses vary in different scenarios, which seems unlikely to be simply attributed to certain factors. The non-linear responses can also be observed in other studies such as Khoi and Suetsugi (2014) [5], Nie et al. (2011) [20] and Park et al. (2011) [60], in which land use and climate changes were shown to exert varying degrees of impacts to hydrological components among different scenarios and time periods. Besides the inherent highly nonlinear hydrological processes, the temporally and spatially varying changes in land use and climate in sub-basins jointly contribute to the non-linear hydrological responses over the entire basin. From Table 10, it can be seen that the wettest and mild warming climate without land use changes, i.e., Wettest and Warm scenario, causes the largest increase in streamflow; and the wettest and extremely warming climate with land use changes, i.e., LUCC & Wettest and Warmest scenario, induces the largest increase in ET. This is because, as reported by Jiao et al. (2013) [96] and Sang et al. (2014) [18], the increase of precipitation will rise both streamflow and ET whereas the increase of temperature will rise ET but reduce streamflow in the upper HRB. Meanwhile, the projected land use changes tend to increase ET and simultaneously decrease streamflow.

Compared with the baseline scenario, the variations in ET and streamflow are proven to be significant at a 5% significance level (α = 0.05), while the changes in surface runoff are significant at a 10% significance level (α = 0.1), under all the scenarios. The changes in groundwater discharge are significant at a 5% significance level (α = 0.05) under all the scenarios except Wet and Warmest, and LUCC & Wet and Warmest. It can be concluded that the hydrological regimes in the near future are potentially significantly affected by land use change and climate variability.

The streamflow at the watershed outlet is projected to increase in the context of climate and land use changes, which is consistent with the findings documented in other studies [23, 30, 39]. In other words, more surface water resources would be available to supply the middle HRB where the population is concentrated and the water consumption is very high. However, it is not completely optimistic. The farmland in the middle HRB at the same time is also expected to increase, owing to the rising population and growing economy [25, 26]. As a result, the demands for water resources will possibly become much stronger than before. The situation of overexploitation of water resources which already exists will be more severe and will inevitably lead to competition against ecological use of water [26]. Hence, from the perspective of integrated management of river basin, proper controlling of farmland expansion and groundwater overdraft still needs to be strengthen in the middle HRB, even with rising streamflow discharges from the upper HRB. Furthermore, it should be aware that the increasing streamflow may also be along with more frequent flash floods and consequently more risks of flood hazard [24].

### 3.5 Limitations and uncertainties

Three models including Markov chain, Dyna-CLUE and SWAT have been implemented in this study. The modeling process which involves complex model settings is likely to bring uncertainties to the results. Besides, the model choice and the model themselves, especially the physics-based hydrological model, are important sources of uncertainty [1, 73]. All these related issues need to be further investigated and quantitatively assessed.

The Markov chain and Dyna-CLUE models were combined in this study to project the spatiotemporal patterns of land use change in the future. But, in principle, the Markov chain is based on a certain type of trend extrapolation. The method is therefore under the assumption that projected land use change will follow historical trend. To examine its applicability, we validated the combined models against three available actual land use maps for the years 1986, 2000 and 2011, as presented in Section 3.1.1. The results demonstrated that the approach is reliable and can be used to capture the trend in land use, as stated in other studies [57, 61, 62]. Nevertheless, it is hard to ensure the projected changes will be realistic for the future because the changes are controlled by many incentives such as economic development, land use policy, and natural environment factors which, however, were not fully considered [97]. Thus, the projected land use pattern in 2024 in this study only represents one possible scenario which assumes that future land use change will follow the trend described by the actual land use patterns in 2000 and 2011, and the interpretation of the results should not be beyond this assumption.

This study attempts to disentangle potential effects of land use change and climate variability in the near future by specifically designing various scenarios of land use and climate changes, as shown in Section 2.5.2. One implicit assumption behind the scenarios settings is that land use change is independent of climate variability. However, they are actually inter-related with each other [17]. Climate change may influence the land use conversions through physical feedback and human adaptation. Conversely, the land use change such as urbanization and forest degeneration may result in climate variations. The mutual impacts, which may lead to hydrological variations, are difficult to examine and were not considered in the present study. Further studies should be conducted to incorporate interactions between land use change and climate variability into the designed scenarios for more reliable assessments of hydrological impacts.

The hypothetical climate change scenarios with varying warmness and wetness were established by referring to the long-term observations of climate variations from 1960–2014. These scenarios can typically represent a range of climatic conditions in the near future. However, because the scenario data of precipitation and temperature for the near future were generated by uniformly adjusting the historical records over the period 2005–2014, the climate scenarios inevitably hold some deficiencies. The hypothetical scenario will have the same distribution of wet and dry days as the baseline period, which, however, should not be real. Moreover, because the same percentage increase in precipitation was applied to each month, the seasonality of precipitation changes was not taken into consideration, which might lead to biases in the results, since climate change might affect differently in different seasons [5, 98], especially in the upper HRB with strong climatic seasonality. In addition, the hypothetical scenario is insufficient in reproducing some extreme climate events that likely take place in the future. But in view of our purpose, which is not to accurately predict climate changes but to assess the potential hydrological impacts within a range of reasonable possibilities, the adopted climate scenarios are applicable in this study and can provide valuable and inspiring information for local water resources management, planning and impact mitigation.

## Conclusions

This study assessed the separate and combined hydrological impacts of land use change and climate variability in the headwater region of the Heihe River Basin, a typical inland river basin in the arid northwest China, in the recent past (1995–2004) and near future (2015–2024), by applying combined land use change models (Markov chain and Dyna-CLUE) and a spatially distributed hydrological model (SWAT). During the period from 1995 to 2014, land use changes in the study area are dominated by the increase in grassland area and the decrease in farmland area. The climate in this period presents a wetting and warming trend. The hydrological modeling reveals that land use changes in the past cause slight reductions in surface runoff, groundwater discharge and streamflow and climate changes conversely induce pronounced increases in them owing to remarkably rising precipitation. As a result of joint effects of land use change and climate variability, the surface runoff, groundwater discharge, ET and streamflow are increased by 18.29%, 11.53%, 9.47% and 11.96%, respectively. The joint variations are approximately as much as those induced solely by climate changes. Spatially, the effects of land use change and climate variability vary with sub-basin. The influences of land use changes are more identifiable in some sub-basins, compared with those on the basin scale.

The potential hydrological impacts in the near future were also explored using the projected land use pattern in 2024 and the hypothetical climate scenarios established upon the analyses of long-term climate observations (1960–2014). Compared with the baseline scenario which keeps the climate and land use conditions at the 2005–2014 level, slight increase in ET and small declines in surface runoff, groundwater discharge and streamflow were modelled if only land use change is considered. Much obvious increases in surface runoff, groundwater discharge, ET and streamflow, however, are projected if only climate change is involved. When land use and climate changes are simultaneously considered, the combined effects are highly similar to those caused exclusively by climate change.

In both timeframes, the hydrological variations are mainly caused by climate variability rather than by land use change in the study area. Land use changes exert a relatively small influence on the hydrological regimes which offsets the impacts induced by a wetter climate. However, the role of land use change should not be overlooked if the climate becomes drier in the future, as it can magnify the overall hydrological responses. By detecting the separate and the combined effects of land use change and climate variability, this study enhances the understanding of the hydrological variations in an arid inland river basin. Moreover, the scenario-based modeling results depict a range of future hydrological possibilities, which might be valuable and inspiring for local authorities and stakeholders to develop, adapt and optimize land and water resource management in the Heihe River basin as well as the other inland river basins across the world which have similar water problems.
